# Herpes Simplex Virus 1 ICP22 Inhibits the Transcription of Viral Gene Promoters by Binding to and Blocking the Recruitment of P-TEFb

**DOI:** 10.1371/journal.pone.0045749

**Published:** 2012-09-24

**Authors:** Lei Guo, Wen-juan Wu, Long-ding Liu, Li-chun Wang, Ying Zhang, Lian-qiu Wu, Ying Guan, Qi-han Li

**Affiliations:** 1 Institute of Medical Biology, Chinese Academy of Medicine Science, Peking Union Medical College, Kunming, People's Republic of China; 2 The First Affilliated Hospital of Kunming Medical College, Kunming, People's Republic of China; 3 Institute of Materia Medica, Chinese Academy of Medicine Science, Peking Union Medical College, Beijing, People's Republic of China; Queen's University, Canada

## Abstract

ICP22 is a multifunctional herpes simplex virus 1 (HSV-1) immediate early protein that functions as a general repressor of a subset of cellular and viral promoters in transient expression systems. Although the exact mechanism of repression remains unclear, this protein induces a decrease in RNA polymerase II Serine 2 (RNAPII Ser-2) phosphorylation, which is critical for transcription elongation. To characterize the mechanism of transcriptional repression by ICP22, we established an *in vivo* transient expression reporter system. We found that ICP22 inhibits transcription of the HSV-1 α, β and γ gene promoters. The viral tegument protein VP16, which plays vital roles in initiation of viral gene expression and viral proliferation, can overcome the inhibitory effect of ICP22 on α-gene transcription. Further immunoprecipitation studies indicated that both ICP22 and VP16 bind to positive transcription elongation factor b (P-TEFb) and form a complex with it *in vivo*. We extended this to show that P-TEFb regulates transcription of the viral α-gene promoters and affects transcriptional regulation of ICP22 and VP16 on the α-genes. Additionally, ChIP assays demonstrated that ICP22 blocks the recruitment of P-TEFb to the viral promoters, while VP16 reverses this blocking effect by recruiting P-TEFb to the viral α-gene promoters through recognition of the TAATGARAT motif. Taken together, our results suggest that ICP22 interacts with and blocks the recruitment of P-TEFb to viral promoter regions, which inhibits transcription of the viral gene promoters. The transactivator VP16 binds to and induces the recruitment of P-TEFb to viral α-gene promoters, which counteracts the transcriptional repression of ICP22 on α-genes by recruiting p-TEFb to the promoter region.

## Introduction

Herpes simplex virus 1 (HSV-1) is a member of the herpes virus family and has a 152 kb double-stranded DNA genome that encodes for more than 80 proteins and possesses complex transcriptional regulation mechanisms [1]–[2]. During lytic and latent infection, some viral proteins engage in viral transcriptional processes via interactions with host cell molecules [3]–[4]. During viral replication, three gene classes, known as the immediate early genes (α-genes), the early genes (β-genes), and the late genes (γ-genes), are sequentially expressed following the comprehensive transcriptional regulation of viral and host cell proteins [5]–[7]. During this process, the viral tegument protein VP16 and the host proteins HCF-1 and Oct-1, potentially in association with other viral proteins, initiate the transcription of the α-genes [8]. The viral immediate-early proteins, including ICP0, ICP4, ICP27, and ICP22, are induced next, and these proteins may be involved in the transcriptional regulation of β- and γ-genes and the feedback regulation of α-genes, though the mechanisms regulating these processes are not completely understood [9]–[12].

VP16, in association with the host cellular proteins Oct-1 and HCF-1, recognizes the core motif TAATGARAT in α-gene promoter sequences, thus initiating transcription of the α-genes [13]–[14]. We have previously shown that the specific function of VP16 on this DNA sequence can overcome the inhibitory effect of ICP22 on α-gene transcription in an *in vivo* transient expression system [15]. The immediate early protein ICP22, which may have multiple functions in viral proliferation [11], inhibits the transcription of many cellular and viral gene promoters in transient expression system [15]. Studies performed by Prod'hon and Bowman confirmed these results [16]–[17]. The data also indicate that this inhibitory effect is not affected by cellular trans-factors that act on gene promoter sequences within the *in vivo* transient expression system [15]. However, despite lacking the ability to bind to specific DNA elements, several studies have shown that ICP22 can interact with cellular transcriptional regulation-related proteins [18]–[20]. Importantly, ICP22 can modulate the level of phosphorylation of the second serine (Ser-2) in the carboxyl-terminal domain (CTD) repeats of the large subunit of RNA polymerase II (RNAPII), which functions in viral gene transcription [7], [20]–[22]. RNAPII is a 12-subunit multi-protein complex that functions in mammalian cell transcription [23]. Among these subunits, there are 52 repeats of a 7 amino acid sequence (YSPTSPS) within the CTD of the large subunit (LS) [24]. The phosphorylation of Ser-2 and Ser-5 of this peptide directly affects RNAPII activity [25]. The phosphorylation of Ser-5 is downstream of Cdk7, the kinase of the RNAPII complex TFIIH [26]. Moreover, Ser-2 is phosphorylated by Cdk9, the kinase of the positive transcription elongation factor complex P-TEFb [27]. The phosphorylation and dephosphorylation of the CTD are dynamic processes within the transcription cycle and are regulated by P-TEFb [22]–[28]. P-TEFb, as described with various data, is composed of cyclin-dependent kinase 9 (CDK9) and its regulatory partner cyclin T1 [27]. As like any CDK-cyclin pairs, CDK9 exerts its kinase activity only when associated with its cyclin partner [29]. The recent studies suggest that P-TEFb is capable of impacting multiple steps in gene expression, from transcription elongation and co-transcriptional control of mRNA processing and export through the CTD, to mRNA translation in the cytoplasm [30]. There are evidences indicating a subset of promoter-bound transcription activators can recruit P-TEFb to the genes in a specific manner of interacting with it, which probably include Brd4, Tat of HIV, the 5′-cap methyltransferase, and H3S10P [31]–[34]. Principally, the obtained data suggest a basic model as below. At the initiation of transcription, hypophosphorylated RNAPII (RNAPII_A_) is bound to elements within the promoter region. After the phosphorylation of Ser-5, the transcription complex enters into the early extending mode. Soon after, transcription of the mRNA molecule resulting from the early extending mode is terminated by two negative elongation factors, DSIF and NELF, which associate with the transcription complex. The next step is capping of the transcript by the capping enzyme. After the capping process, P-TEFb functions as a key factor to ensure that the transcription complex resumes normal elongation. The phosphorylation of Ser-2 of the CTD converts RNAPII into the elongation active hyperphosphorylated state (RNAPIIo). Simultaneously, P-TEFb also phosphorylates the SPT5 and RD subunits of the two negative elongation factors DSIF and NELF, respectively. The phosphorylation of these two subunits is a key step in the dissociation of these two negative elongation factors from the transcription complex. Thus, RNAPII drives the transcription complex into the normal elongation state, which is a very important event during transcription [35]–[36]. Fraser *et al.* suggest that during HSV-1 infection ICP22 triggers a decrease in RNAPII Ser-2 phosphorylation and induces an intermediate status (RNAPII_I_), which causes abnormalities in cellular-related gene transcription and optimal expression of viral genes [21], [37]–[40].

We used an *in vivo* transient expression reporter system and HSV1-infected cells to add to our previous studies that indicated a transcriptional co-regulation effect of ICP22 and VP16 on viral α-genes. We observed that ICP22 represses transcription of the viral α-, β- and γ-gene promoters by interacting with and blocking the recruitment of P-TEFb to promoter regions. In contrast, VP16 overcomes the transcriptional inhibition of ICP22 by binding to and recruiting P-TEFb to promoter regions containing the TAATGARAT motif such as α-genes. Thus, based on this model, the current paper suggested a possible manner of recruitment of P-TEFb to the promoter of HSV1 α-gene in viral infection, in which ICP22 and VP16 may regulate P-TEFb through binding the different components of it.

## Results

### Transcription regulation of the HSV-1 α-, β- and γ-gene promoters by ICP22 and VP16 in an *in vivo* dual luciferase reporter system

**Figure 1:**
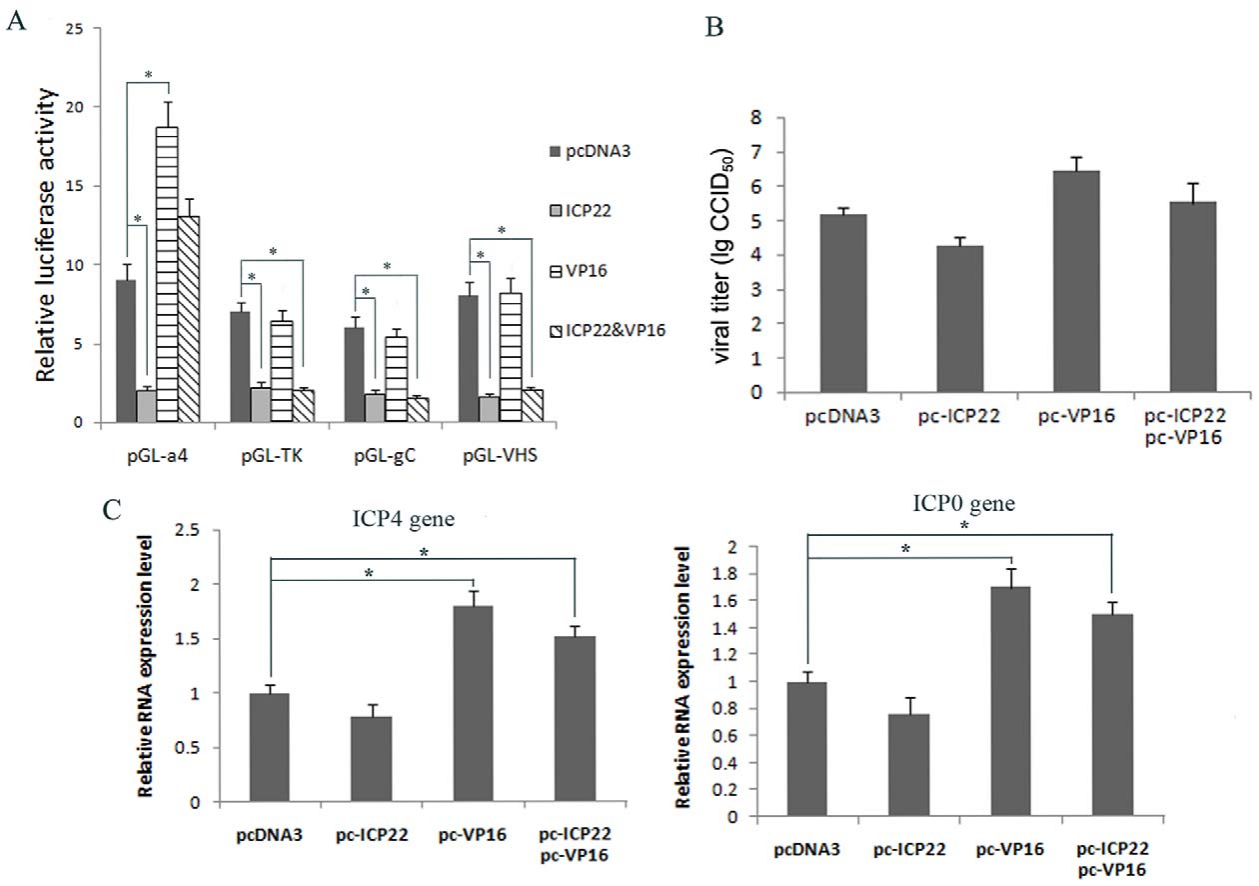
ICP22 and VP16 co-regulate transcription of HSV-1 gene promoters and viral proliferation. (A) The transcriptional regulation of HSV-1 α-, β- and γ-gene promoters by ICP22 and VP16. CHO-K1 cells were co-transfected with the reporter plasmids (pGL-α4, TK, gC, or VHS) and the effector expression plasmids (pcDNA-ICP22 and/or pcDNA-VP16) as indicated for 36 h, each group took transfection of pcDNA3 as a control. The cell lysates were analyzed by the dual luciferase assay and normalized with the renilla luciferase expression. (B, C) Increasing the expression levels of ICP22 and VP16 affects viral proliferation and α-gene expression. Hep-2 cells were co-transfected with ICP22, VP16 expression plasmids as indicated (pcDNA3 as control), all transfections were balanced to a total equal amount of DNA with pcDNA3. At 30 h post-transfection, the cells were infected with HSV-1 at MOI of 1.5 (B) or MOI of 10 (C). At 4 h post-infection, total RNA was extracted from the cell lysates (C) and subjected to quantitative RT-PCR to measure the RNA level of viral ICP4 and ICP0 segments with β-actin RNA serving as an internal control. At 20 h post-infection, the medium from Hep-2 cells (B) was collected to measure the virus titer by microtitration assay. Error bars represent the standard deviation from triplicate samples. * P<0.05 by student's t-test.

Although the immediate early proteins are involved during the regulation of viral gene transcription in the HSV-1 infection process, the function of ICP22 is not yet clear. Studies have shown that ICP22 is involved in viral gene transcriptional regulation through multiple mechanisms and that this protein can be observed in the transcriptional complex of HSV-1 infected cells [19], [41]–[42]. Prod'hon *et al.* have reported that ICP22 can inhibit the transcription of several genes [17], this observation is especially noteworthy in HSV-1 α-gene studies [15]–[16]. To investigate the effect of ICP22 on transcriptional inhibition, we established an *in vivo* transient expression system using the dual luciferase reporter in which pRL-CMV, the internal transfection control reporter plasmid, and pcDNA3, the eukaryotic expression vector used to express ICP22 and other proteins, are both driven by the CMV IE promoter. The activity of this promoter in the presence of ICP22 had been confirmed in our previous study [15]. Using this reporter system, we studied the inhibition effect of ICP22 on viral gene transcription and the relationship between ICP22 and VP16 in this transcriptional regulation. The results suggest that ICP22 inhibits transcription from the viral promoters of the α-, β- and γ-genes (Figure 1A). Interestingly, this inhibitory effect of ICP22 on the α4 promoter is diminished when VP16 is present (Fig. 1A). However, VP16 does not have the same effect on transcription of the β- and γ-genes, such as the TK, gC and VHS genes (Fig. 1A). To confirm this observation from the transient expression system, HSV-1 titers in Hep-2 cells transfected previously with pcDNA-ICP22 and/or pcDNA-VP16 were detected by microtitration assay. These results suggested that increased levels of ICP22 repressed viral proliferation and that increased levels of VP16 promoted viral proliferation and released the repressing effect of ICP22 (Fig. 1B). Further, the detection of ICP4 and ICP0 mRNA by q-RT-PCR in the infected cells with increased expression of ICP22 and/or VP16 suggested the same effects (Fig. 1C). ICP22 had an inhibitory effects on ICP4 and ICP0 expression, which was overcome by the expression of VP16, indicating that the effects of ICP22 and VP16 on viral proliferation might be mediated by effects on viral transcription (Fig. 1C).

### The interactions of ICP22 and VP16 with P-TEFb

**Figure 2:**
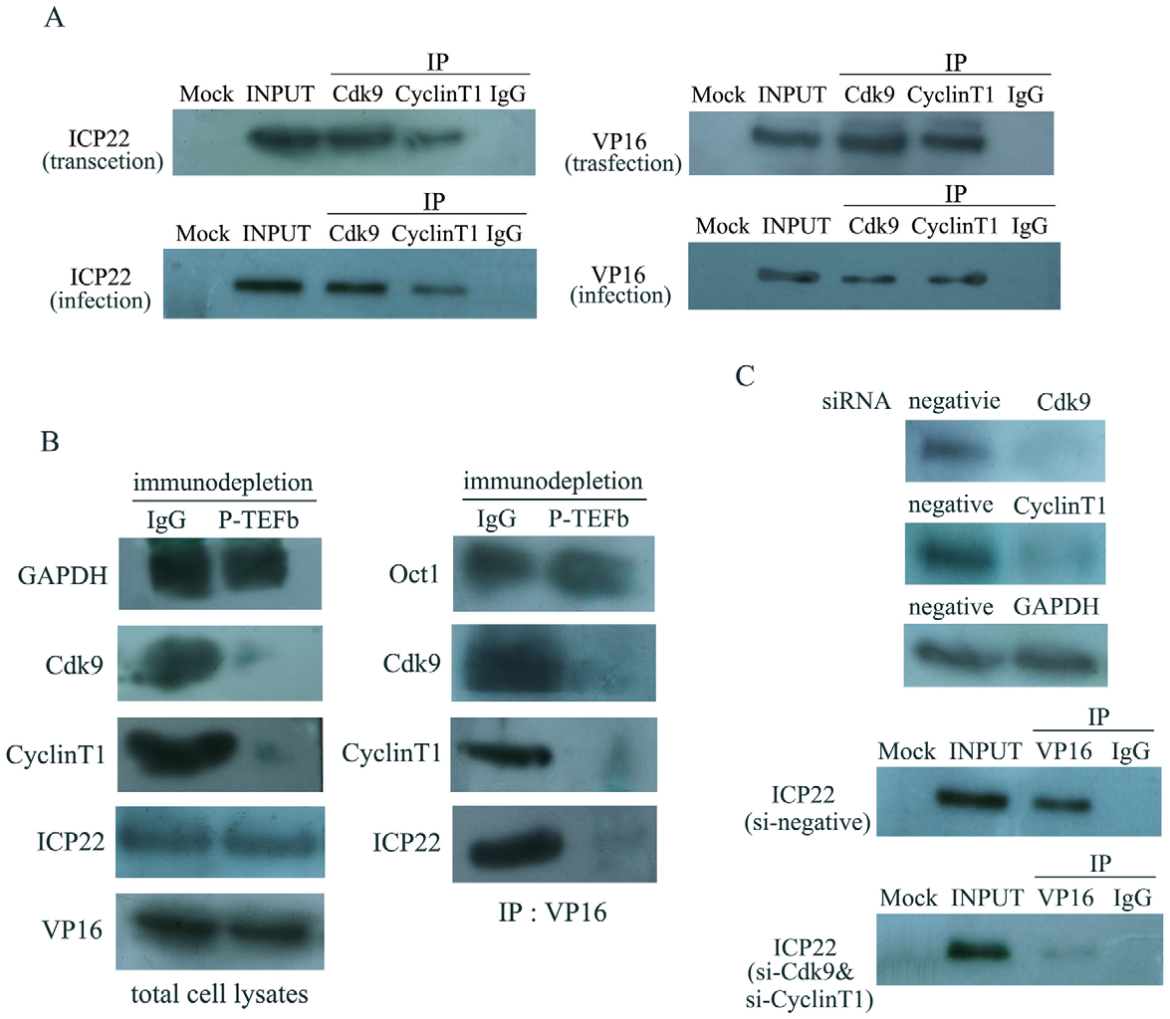
The interactions between VP16, ICP22 and P-TEFb *in vivo*. (A) Co-immunoprecipitation of interaction between ICP22 and VP16 *in vivo*. CHO-K1 cells were co-transfected with pcDNA-ICP22 and pcDNA-VP16 plasmids, or Hep-2 cells were infected with HSV-1 (MOI = 1) for 40 h. In each case the cell lysates were immunoprecipitated with mouse anti-ICP22 or rabbit anti-VP16 polyclonal antibody, or mouse/rabbit IgG as a control, and immunoblotted with ICP22 or VP16 polyclonal antibody. (B) Co-immunoprecipitation of interactions between ICP22 and P-TEFb, VP16 and P-TEFb *in vivo*. CHO-K1 cells were transfected with pcDNA-ICP22 and pcDNA-VP16 plasmids, or Hep-2 cells were infected with HSV-1 (MOI = 1) for 40 h. In each case the cell lysates were immunoprecipitated with rabbit anti-Cdk9 and anti-CyclinT1 polyclonal antibodies (rabbit IgG as a control) and immunoblotted with ICP22 or VP16 polyclonal antibody. (C) Co-immunoprecipitation of interaction between ICP22 and VP16 via immunodepletion of P-TEFb. CHO-K1 cells were co-transfected with pcDNA-ICP22 and pcDNA-VP16 plasmids for 40 h. The cell lysates were immunodepleted with Cdk9 and CyclinT1 antibodies or rabbit IgG (control) before following procedure, the equal amounts of the immunodepleted cell lysates immunoblotted with the indicated antibodies (left panel) or immunoprecipitated with anti-VP16 beads followed by immunoblotting with the indicated antibodies (right panel). (D) The effects of Cdk9 and CyclinT1 RNA interference analyzed by western blotting and co-immunoprecipitation of interaction between ICP22 and VP16 via knocking down P-TEFb. Hep-2 cells were transfected for 40 h with si-Cdk9, si-CyclinT1 or the scrambled interfering RNA as a negative control at a concentration of 100 nM. Equal amounts of the cell lysates were immunoblotted with Cdk9, CyclinT1 or GAPDH polyclonal antibodies (upper panel). Hep-2 cells were co-transfected with the specific Cdk9 and CyclinT1 siRNA or the scrambled negative siRNA (100 nM) for 12 h, then infected with HSV-1 (MOI = 1). At 36 h after infection, equivalent amounts of protein from the whole cell lysates were immunoprecipitated with the rabbit anti-VP16 polyclonal antibody or rabbit IgG as a control, and immunoblotted with the ICP22 polyclonal antibody (lower panel).

Because the functional association between VP16 and ICP22 on transcriptional regulation of HSV-1 α-genes, we examined the possible interaction between ICP22 and VP16 using *in vivo* and *in vitro* binding assays. Despite not being detected by yeast trap experiment and *in vitro* binding assay (Fig. S1), there is an interaction between ICP22 and VP16 in cells co-transfected with ICP22 and VP16 and, importantly, in HSV-1 infected cells, which we detected by co- immunoprecipitation (Fig. 2A). Because ICP22 and VP16 can interact with various cellular proteins in infected or transfected cells [11]–[14], and recent *in vitro* studies suggested that ICP22 and VP16 interact with components of the P-TEFb complex Cdk9, and CyclinT1, respectively [20], [43], we examined the interactions between ICP22 and VP16 with P-TEFb by co-immunoprecipitation and immunodepletion. First, we detected the interactions of P-TEFb with ICP22 and VP16 by co-immunoprecipitating CHO-K1 cells co-transfected with pcDNA-ICP22 and pcDNA-VP16 plasmids for 40 h with Cdk9 and CyclinT1 antibodies, respectively, the interactions were confirmed in HSV-1 infected cells. Immunoblot analysis with ICP22 and VP16 antibodies suggested that ICP22 and VP16 are capable of interacting with P-TEFb (Fig. 2B). We further analyzed the interaction between ICP22 and VP16 using an immunodepletion assay to test if the interaction is mediated by the P-TEFb complex. The results confirmed these interactions because specific depletion of Cdk9 and CyclinT1 inhibited almost all of the interactions between ICP22 and Cdk9, VP16 and CyclinT1, and ICP22 and VP16 (Fig. 2C). Finally, we evaluated these interactions during HSV-1 infection in Hep-2 cells co-transfected with si-Cdk9 and si-CyclinT1 siRNAs to knockdown P-TEFb complex expression (Fig. 2D). Immunoprecipitation with the VP16 antibody, followed by the immunoblotting with the ICP22 antibody, indicated that knockdown of P-TEFb complex expression decreased the interaction between ICP22 and VP16 (Fig. 2D). Taken together, these results indicated that both ICP22 and VP16 are capable of interacting with P-TEFb and thus form a complex through their mutual association with the components of P-TEFb.

### Changes in the expression level of the components of P-TEFb affect transcriptional inhibition by ICP22 and the blocking of transcriptional inhibition by VP16

**Figure 3:**
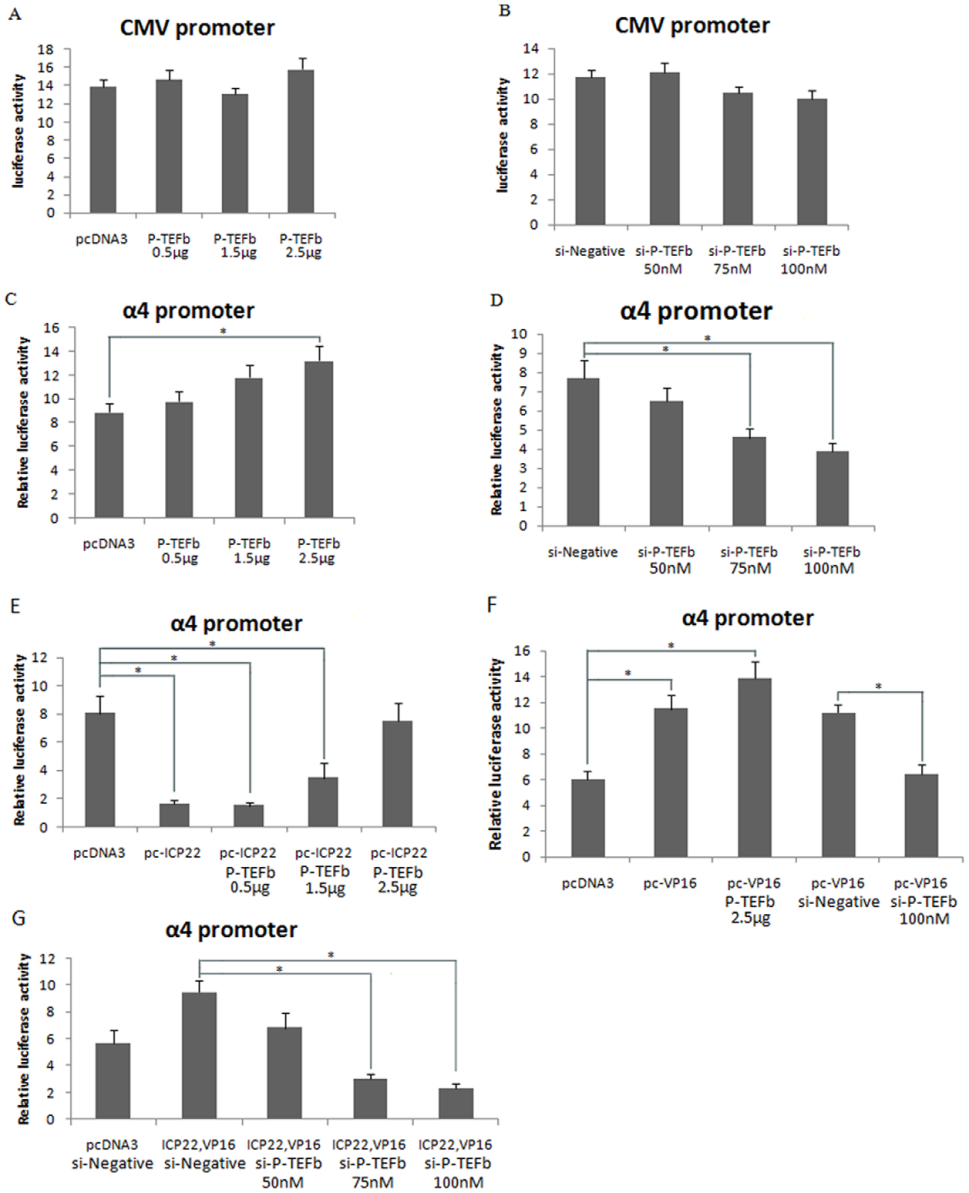
The expression level of P-TEFb affects the transcriptional regulation of the α-gene promoter by ICP22 in association with VP16. (A, B) Transcriptional activity of the CMV promoter with changing levels of P-TEFb expression. CHO-k1 or Hep-2 cells were co-transfected for 36 h with the pRL-CMV reporter plasmid and P-TEFb expression plasmids (pcDNA-Cdk9 and pcDNA-CyclinT1, pcDNA3 as a control) (A) or siRNA against P-TEFb (si-Cdk9 and si-CyclinT1, si-Negative RNA as a control) (B) as indicated, then the cell lysates were analyzed with the luciferase assay. (C, D) Transcriptional activity of the viral α4 gene promoter with changing levels of P-TEFb expression. CHO-k1 or Hep-2 cells were co-transfected for 36 h with pGL-α4 reporter plasmid and P-TEFb expression plasmids (pcDNA-Cdk9 and pcDNA-CyclinT1, pcDNA3 as a control) (C) or siRNA against P-TEFb (si-Cdk9 and si-CyclinT1, si-Negative RNA as a control) (D) as indicated, then the cell lysates were analyzed by the dual luciferase assay and normalized with the renilla luciferase expression. (E) P-TEFb overcomes transcriptional inhibition of the α4 promoter by ICP22 via increasing the expression level. CHO-K1 cells were co-transfected for 36 h with pGL-α4 reporter plasmid and ICP22, P-TEFb (Cdk9 and CyclinT1) or pcDNA3 control expression plasmids, as indicated. The cell lysates were then analyzed by the dual luciferase assay and normalized with the renilla luciferase expression. (F) The expression level of P-TEFb affects transcriptional activation of the α4 promoter by VP16. Hep-2 cells were co-transfected for 36 h with pGL-α4 reporter plasmid and VP16 expression plasmid, together with P-TEFb expression plasmid (pcDNA-Cdk9 and pcDNA-CyclinT1, pcDNA3 as a control) or siRNA against P-TEFb (si-Cdk9 and si-CyclinT1, si-Negative RNA as a control), as indicated. The cell lysates were then analyzed with the dual luciferase assay and normalized with the renilla luciferase expression. (G) Decreased expression of P-TEFb influences the ability of VP16 to release transcriptional suppression of the α4 promoter by ICP22. Hep-2 cells were co-transfected for 36 h with pGL-α4 reporter plasmid, expression plasmids (ICP22 and VP16, pcDNA3 as a control) and siRNA against P-TEFb (si-Cdk9 and si-CyclinT1, si-Negative RNA as a control), as indicated. The cell lysates were then analyzed with the dual luciferase assay and normalized with the renilla luciferase expression. Error bars represent the standard deviation from triplicate samples. * P<0.05 by student's t-test.

Because P-TEFb is an important transcriptional control factor in the RNAPII complex, a change in P-TEFb expression can directly affect the efficiency of both cellular and viral gene expression [27], [37], [44]. To confirm whether ICP22 and VP16 influence viral α-gene transcription via interactions with the components of P-TEFb, we designed our study to measure the effects of changes in the level of components of P-TEFb expression including that of CDK9 and CyclinT1. To ensure accurate results, the transcriptional activity of the CMV promoter was measured using a luciferase activity assay in cells with increased or decreased levels of P-TEFb expression. The results indicate that changes in the level of P-TEFb do not impact the transcriptional activity of the CMV promoter (Fig. 3A, B). Second, we analyzed whether P-TEFb regulates transcription of the HSV-1 α4 promoter in a transient expression system *in vivo* in cells with increased or decreased levels of P-TEFb expression. The results indicate that the transcriptional activity of the α4 promoter is affected by the expression level of P-TEFb (Fig. 3C, D). Based on these observations, further experiments were conducted to investigate the effects of the P-TEFb expression level on the inhibition of α4 promoter transcription by ICP22 and the release of this effect by VP16. The results show that the transcriptional inhibition by ICP22 diminishes gradually as the expression of P-TEFb increases in CHO-K1 cells co-transfected with pGL-α4, pcDNA-ICP22, pcDNA-Cdk9 and pcDNA-CycinT1 (Fig. 3E). In contrast, the transcriptional activation of the α4 promoter by VP16 is diminished as the P-TEFb expression level decreases in CHO-K1 cells co-transfected with pGL-α4, pcDNA-VP16, si-Cdk9 and si-CyclinT1 (Fig. 3F). Finally, the ability of VP16 to release the inhibition of viral α4 promoter transcription by ICP22 is reduced as the expression level of P-TEFb decreased in CHO-K1 cells co-transfected with pGL-α4, pcDNA-VP16, pcDNA-ICP22, si-Cdk9 and si-CyclinT1 (Fig. 3G). Taken together, these results suggest that the transcriptional inhibition of α-genes by ICP22 and the release of this inhibition by VP16 are both dependent on P-TEFb.

### ICP22 represses transcription of HSV-1 gene promoters by inhibiting the recruitment of P-TEFb to the promoter regions

**Figure 4:**
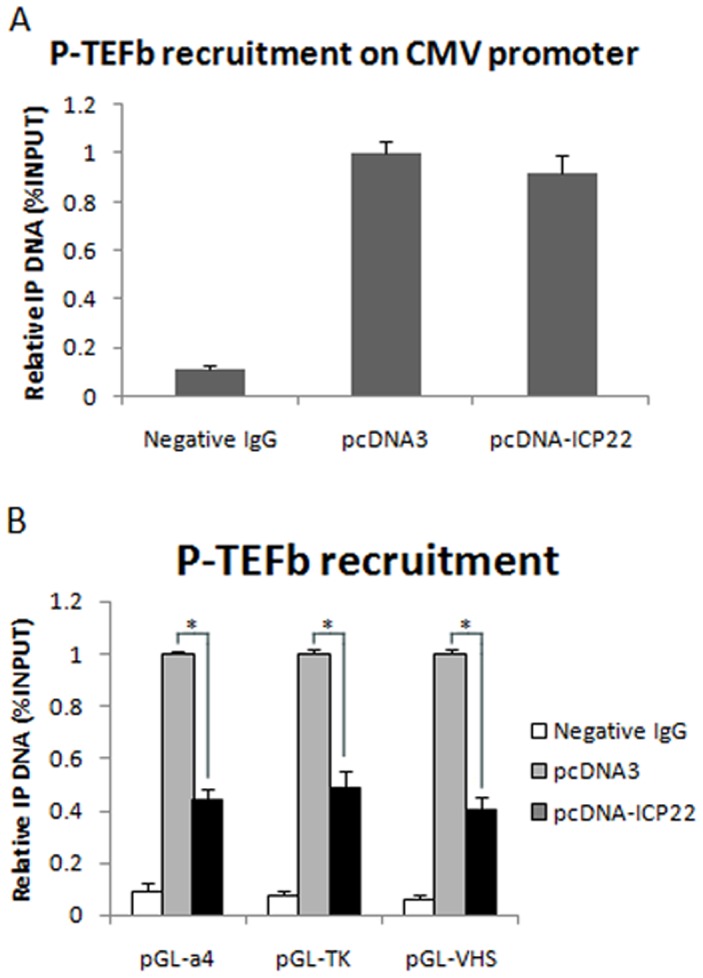
ICP22 inhibits the recruitment of P-TEFb to HSV-1 gene promoter regions. (A) The effect of ICP22 on recruitment of P-TEFb to the CMV IE promoter. CHO-K1 cells were co-transfected for 40 h with pRL-CMV plasmid containing the CMV IE promoter and ICP22 expression plasmid or pcDNA3 as a control, then subjected to ChIP assays. (B) ICP22 inhibits the recruitment of P-TEFb to promoter regions of HSV-1α-, β- and γ-genes. CHO-K1 cells were co-transfected for 40 h with reporter plasmids containing α4, TK or VHS promoter and ICP22 expression plasmid or pcDNA3 as a control as indicated and subjected to ChIP assays. Antibodies specific for CyclinT1 or control rabbit IgG were used for immunoprecipitation. The precipitated DNA was analyzed by RT-PCR using primers specific for the promoter regions of CMV IE, HSV-1 α4, TK, and VHS. The values are the percentage of immunoprecipitated input DNA relative to the control group transfected with pcDNA3. Error bars represent the standard deviation with triplicate samples. * P<0.05 by student's t-test.

P-TEFb need to be recruited to the promoter region of transcription initiation to enhance transcription elongation through the phosphorylation of RNAPII CTD Ser-2, while ICP22 can induce abnormal RNAPII CTD Ser-2 phosphorylation by interacting with P-TEFb [20]–[21], [36], [44]. Based on these findings and the data obtained in this study, we performed ChIP experiments using a CyclinT1 antibody to investigate the effect of ICP22 on the recruitment of P-TEFb to the promoter regions of the viral α-, β- and γ-genes. In this experiment, the cross-linking agents EGS and FA were used to enhance the protein-protein and protein-DNA interactions (Fig. S2). To show that the ICP22-mediated recruitment of P-TEFb is specific to the viral α-, β- and γ-gene promoters, we first analyzed the effect of ICP22 on P-TEFb recruitment to the CMV IE promoter using the ChIP assay. The results confirm that cellular expression of ICP22 does not impact the recruitment of P-TEFb to the CMV IE promoter (Fig. 4A). Next, the ChIP assay was conducted with a CyclinT1 antibody to determine the effect of ICP22 on P-TEFb recruitment to the viral α-, β- and γ-gene promoters. This experiment shows that in the presence of ICP22, the amount of P-TEFb recruited to the promoter regions of the viral α-, β- and γ-genes is clearly decreased (Fig. 4B). Altogether, the results suggest that the interaction between ICP22 and P-TEFb inhibits the recruitment of P-TEFb to the viral gene promoter region and reduces the efficacy of viral transcription, potentially by inducing the intermediate status of RNAPII CTD phosphorylation (RNAPII_I_).

### VP16 releases ICP22 transcriptional inhibition by recruiting P-TEFb to the α-gene promoter region

**Figure 5:**
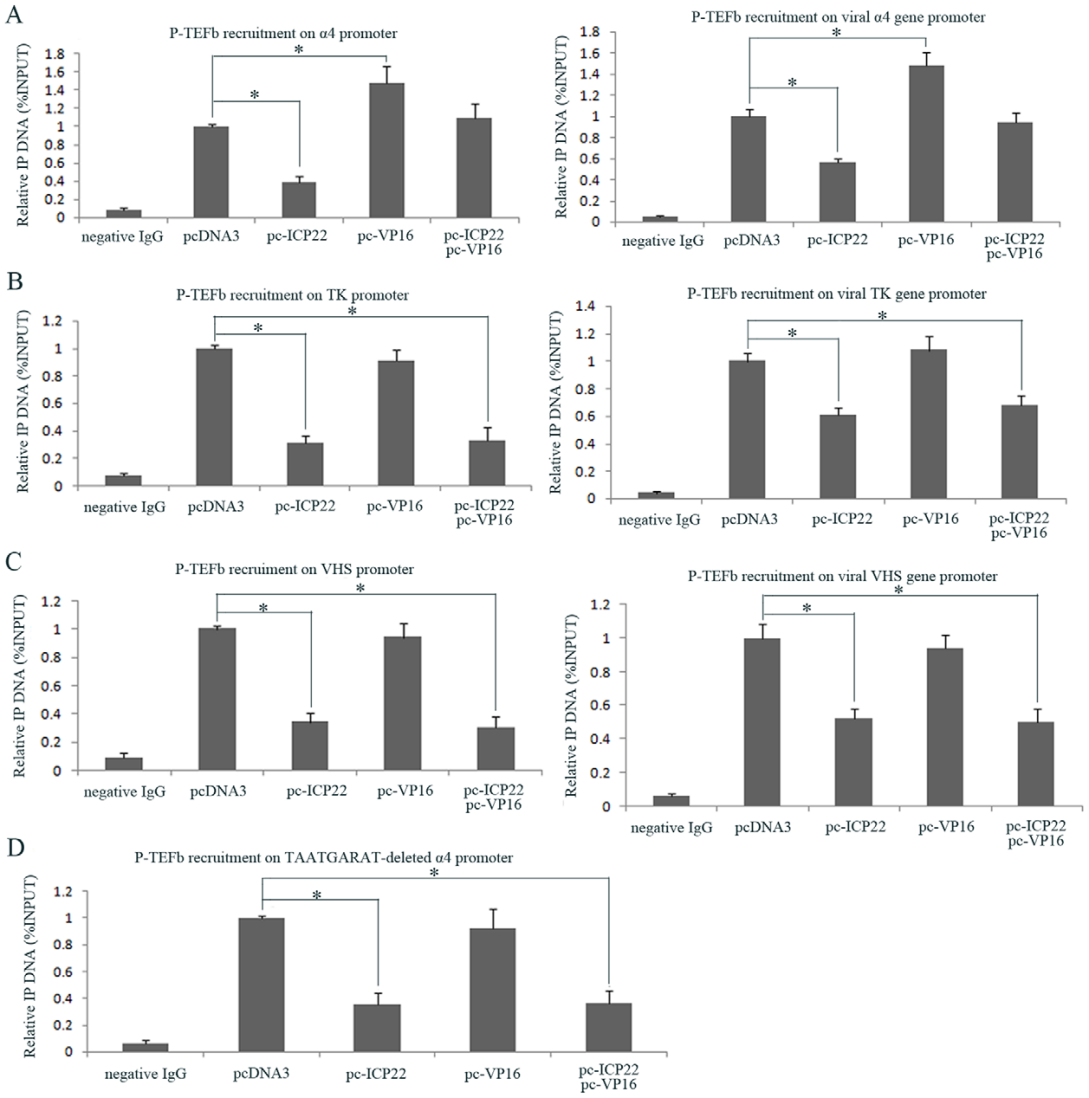
The effects of P-TEFb recruitment on viral α-, β- and γ-gene promoters in the presence of VP16 and ICP22. (A) The effects of VP16 and ICP22 on the recruitment of P-TEFb to viral α4 gene promoter region. CHO-K1 cells were co-transfected with pGL-α4 plasmid containing α4 gene promoter and ICP22, VP16 or control pcDNA3 expression plasmids as indicated for 40 h and subjected to ChIP assays (left panel). Hep-2 cells were transfected with ICP22, VP16 or control pcDNA3 expression plasmids as indicated for 36 h, and then infected with HSV-1 at an MOI of 10. At 3 h post-infection, the cells were subjected to ChIP assays (right panel). (B) The effects of VP16 and ICP22 on the recruitment of P-TEFb to viral TK gene promoter region. CHO-K1 cells were co-transfected with pGL-TK plasmid containing TK gene promoter and ICP22, VP16 or control pcDNA3 expression plasmids as indicated for 40 h and subjected to ChIP assays (left panel). Hep-2 cells were transfected with ICP22, VP16 or control pcDNA3 expression plasmids as indicated for 36 h, and then infected with HSV-1 at an MOI of 2. At 7 h post-infection, the cells were subjected to ChIP assays (right panel). (C) The effects of VP16 and ICP22 on the recruitment of P-TEFb to viral VHS gene promoter region. CHO-K1 cells were co-transfected with pGL-TK plasmid contained VHS gene promoter and ICP22, VP16 or control pcDNA3 expression plasmids as indicated for 40 h and subjected to ChIP assays (left panel). Hep-2 cells were transfected with ICP22, VP16 or control pcDNA3 expression plasmids as indicated for 36 h, and then infected with HSV-1 at an MOI of 2. At 13 h post-infection, the cells were subjected to ChIP assays (right panel). (D) The effects of VP16 and ICP22 on the recruitment of P-TEFb to the TAATGARAT-deleted α4 promoter. CHO-K1 cells were co-transfected with pGL-α4-Δ16 plasmid containing TAATGARAT-deleted α4-gene promoter and ICP22, VP16 or control pcNDA3 expression plasmids as indicated for 40 h and subjected to ChIP assays. Antibodies specific for CyclinT1 or control rabbit IgG were used for immunoprecipitation. The precipitated DNA was analyzed by RT-PCR using primers specific for the promoter regions of α4, TK, VHS and TAATGARAT-deleted α4. The values are expressed as the percentage of immunoprecipitated input DNA relative to the control group transfected with pcDNA3. Error bars represent the standard deviation from triplicate samples. * P<0.05 by student's t-test.

Because VP16 can release the α-gene transcriptional inhibition by ICP22 and interact with P-TEFb, we performed ChIP assays to investigate the role of VP16 together with ICP22 in the recruitment of P-TEFb to the viral α-, β- and γ-gene promoters in the co-transfected transient expression system and in HSV-1 infected cells. The results show both in the transient expression system and in infected cells, that VP16 increases the recruitment of P-TEFb to the viral α-gene promoter but not the β- and γ-gene promoters (Fig. 5A–C). This finding may be because the α-gene promoter possesses the TAATGARAT motif, which VP16 specifically recognizes through its bound Oct-1, whereas the β- and γ-gene promoters do not contain this motif. To test if mutation or deletion of this motif decreases the ability of VP16 to recruit P-TEFb to the α-gene promoter, we performed a ChIP experiment in which the viral α4 gene promoter had a deletion of this motif. Interestingly, the results indicated that the recruitment of P-TEFb to the TAATGARAT-deleted α4 gene promoter was not affected by the presence of VP16 (Fig. 5D). Meanwhile, the results also show that the ICP22-mediated decrease in recruitment of P-TEFb to the viral α-gene promoter is overcome by the presence of VP16 (Fig. 5A) but that VP16 could not release the ICP22-induced block in recruitment of P-TEFb to the β-, γ- and TAATGARAT-deleted α4-gene promoter regions (Fig. 5B–D). Taken together, these findings suggest that VP16 overcomes the transcriptional inhibition by ICP22 by increasing P-TEFb recruitment to the viral α-gene promoter, which is mediated through recognition of the TAATGARAT motif by VP16 in association with HCF-1 and Oct-1.

## Discussion

The function of ICP22, an immediate early protein encoded by HSV-1, has been investigated in many studies of viral proliferation, these studies concluded that the protein possesses multiple functions and these functions optimize the expression of HSV-1 late genes, such as γ2 [31], [45]. In contrast, studies characterizing ICP22 function when expressed in isolation indicated that ICP22 acts as a transcriptional inhibitor of several viral gene promoters, such as the HSV-1 α-gene, the SV40 early gene, and the adenovirus E1A gene [15]–[17], [41]. The mechanism and significance of this contradictory transcriptional regulation by ICP22 and the exact role of ICP22 in viral infection has not been identified. Therefore, we further investigated the function of ICP22, together with VP16, in the transcriptional regulation of the viral α-, β- and γ-genes in an in vivo transient expression system and in infected cells. This study provides additional information on the biological role of ICP22 in the viral transcription process and the mechanism that occurs during viral replication. Importantly, the results obtained in this study confirm the role of P-TEFb complex, in association with ICP22 and VP16, in the transcriptional regulation of HSV-1 α-genes and the findings suggest a possible model of α-gene transcriptional regulation by ICP22 and VP16.

The transcription factor P-TEFb performs key functions in the RNAPII complex, it controls the production of mRNA by regulating RNAPII transcriptional elongation via phosphorylation of Ser-2 of the CTD and of the negative elongation factors DSIF and NELF [27], [35]. A crucial step during transcription is that the recruitment of P-TEFb to the transcriptional start site which allows it to phosphorylate RNAPII and the two negative elongation factors, DSIF and NELF. Because Cdk9 and CyclinT1 do not by themselves possess any DNA-binding activity [44], one of the critical functions of many DNA sequence-specific transcription factors such as Brd4 [46], CIITA [47], NF-kB [48], Myc [49]–[50], STAT3 [51], MyoD [52], B-Myb [53], is to recruit P-TEFb to their respective promoter targets, as while the 7SK snRNA and HEXIM1 protein sequester it into an inactive complex [44]. In addition to its role in cellular gene expression, P-TEFb was also found to interact with viral gene products to facilitate viral life cycle progression. For instance, the replication of HIV requires P-TEFb to function as a co-activator of transcriptional expression. In HIV infection, the viral Tat protein is capable of interacting with P-TEFb and recruiting it to the 5′ viral RNA terminus to enhance viral replication [54]. Additionally, the Tax protein of Human T-lymphotropic virus type 1 (HTLV-1) can also recruit P-TEFb to the RNAPII complex to improve viral transcription [55]–[56]. HSV-1, a DNA virus with a complicated genome structure and transcriptional process, depends on the host cellular gene transcription machinery through interactions of viral and host protein molecules [2]. Our results provide the first evidence that HSV-1 employs P-TEFb to promote the transcription of RNAPII on viral α-genes via VP16-mediated recruitment of it to the α-gene promoters. The function of VP16 in viral α-gene transcriptional activation requires two other cellular proteins, Oct-1 and HCF-1, which form a complex with VP16 that recognizes the core motif TAATGARAT to initiate transcription of the α-gene. Therefore, it is possible that P-TEFb is recruited to the α-gene promoters by the VP16 complex during viral infection. This implication is supported by the results showing that VP16 has no effect on P-TEFb recruitment to the β-, γ- and the TAATGARAT-deleted α-gene promoters.

Our findings confirm the observed interactions between ICP22 and Cdk9, VP16 and CyclinT1 *in vivo* [20], [43], and we further demonstrate that ICP22 and VP16 can form a complex with P-TEFb in an *in vivo* transient expression system and in HSV1-infected cells. Because of the function of P-TEFb in the RNAPII complex, the interactions of P-TEFb with ICP22 and VP16 suggest that transcriptional regulation by ICP22 and VP16 is mediated by P-TEFb. Conversely, the interactions between ICP22 and VP16 with P-TEFb imply that the function of P-TEFb may be interfered with. Based on our luciferase reporter system and ChIP experiments, the observation that ICP22 inhibits transcription of the viral α-, β- and γ-genes is likely due to the interaction between ICP22 and P-TEFb that blocks the recruitment and function of P-TEFb at the viral promoter regions. Similarly, the interaction between VP16 and P-TEFb suggests that the overcoming of ICP22-mediated viral α-gene transcriptional inhibition is likely mediated by the recruitment of P-TEFb to the promoter regions via recognition of the TAATGARAT element of α-genes. In this transcriptional regulation model, the interaction with ICP22 may block the regular cellular recruitment pathways of P-TEFb to viral and cellular gene promoters. In contrast, VP16 interacts with P-TEFb and recruits it to the viral α-gene promoters that contain the TAATGARAT motif and overcomes transcriptional inhibition by ICP22 and promotes transcription of IE genes. Thus, the regulation of P-TEFb recruitment by HSV-1 may inhibit transcription of cellular genes through ICP22 and hijack the host transcription complex for viral gene expression through VP16.

Our experiment was initially performed in an *in vivo* transient expression reporter system and then confirmed in virus-infected cells. All results obtained here suggest ICP22 and VP16 in the transcriptional regulation of viral α-gene promoters and reveal the regulation of P-TEFb function by ICP22 and VP16 during viral infection. These data help to create a model that could be used to investigate additional mechanisms of HSV-1 infections.

## Materials and Methods

### Cell lines, viruses, plasmids and reagents

Chinese hamster ovary cells (CHO-k1) acquired from Kunming Cell Bank (KIZ, Kunming, China) were grown in complete Ham's F12 medium (GIBCO) containing 10% bovine serum at 37°C in 5% CO2. Hep-2 and Vero cells (Institute of Medical Biology, CAMS) were grown in Dulbecco's modified Eagle's medium (GIBCO) supplemented with 10% bovine serum at 37°C in 5% CO_2_ [19], [57]. Stocks of wild-type HSV-1 (strain 17) were prepared in Vero cells.

The eukaryotic expression plasmids pcDNA-ICP22, pcDNA-VP16, pGAD-ICP22 and pGBK-VP16 that contained the entire viral ICP22 or VP16 gene sequence, were generated by our laboratory as previously described [15]. The plasmids pcDNA-Cdk9 and pcDNA-CyclinT1 were constructed by inserting the Cdk9 or CyclinT1 gene sequence into the pcDNA3 vector (Invitrogen). The Cdk9 and CyclinT1 genes were amplified from Hela cell total RNA by reverse transcription PCR (Table S1). Five reporter plasmids, pGL-α4 (α-gene), pGL-α4-Δ16, pGL-TK (β-gene), pGL-gC (γ-gene), and pGL-VHS (γ-gene) were generated by fusing the ICP4 gene promoter (nt −332 to +30), the ICP4 gene promoter with the TAATGARAT motif deleted (nt −244 to +30) [15], the TK gene promoter (nt −250 to +1), the gC gene promoter (nt −124 to +71) and the UL41 (VHS) gene promoter (nt −360 to +40) to the Photinus pyralis luciferase gene in the pGL3-basic vector (Promega).

A rabbit anti-cyclinT1 antibody (sc-10750), a rabbit anti-Cdk9 antibody (sc-8338), a mouse anti-Oct-1 antibody (sc-8024), and control pre-immune rabbit or mouse IgGs were purchased from Santa Cruz Biotechnology. The rabbit anti-VP16 polyclonal antibody (V4388) was purchased from Sigma. The mouse anti-GAPDH monoclonal antibody (AG019) was purchased from Beyotime Biotechnology. The mouse ICP22 antiserum was generated in our lab as previously described [15]. Peroxidase-conjugated goat anti-rabbit and goat anti-mouse antibodies were purchased from Boster (Wuhan, China).

### RNA interference (RNAi) of Cdk9 and CyclinT1 gene expression

The siRNA duplexes against Cdk9 and CyclinT1 were designed according to the sequences by Chiu *et al* (Table S2) [58]. Both the siRNA used for knockdown and the scrambled interfering RNA that was used as the negative control were chemically synthesized with 2′OME modification by GenePharma (Shanghai, China). All siRNAs were stored in 0.1% diethylpyrocarbonatetreated water at −80°C.

Hep-2 cells were seeded in the wells of 6-well plates. On the day after seeding, si-CDK9, si-CyclinT1 or the negative control siRNA was transfected at a final concentration of 100 nM with the Lipofectamine 2000 transfection reagent (Invitrogen) according to the manufacturer's instructions. 40 h after transfection, the transfected cells were harvested, and total cellular protein extracts were prepared for western blot analysis.

### Dual luciferase reporter assays

Luciferase assays were performed using a Dual-Glo luciferase assay system according to the manufacturer's instructions (Promega). Briefly, CHO-k1 cells were cultured in 6-well plates and co-transfected with expression plasmids (ICP22, VP16, Cdk9, and/or CyclinT1) and reporter plasmids (pGL-α4, pGL-TK, pGL-gC or pGL-VHS) with the pRL-CMV plasmid which expresses renilla luciferase as an internal control to normalize the transfection efficiency. For siRNA, Hep-2 cells were co-transfected with siRNA against Cdk9 and CyclinT1 as well as the expression and reporter plasmids mentioned above. All transfections were balanced for a total equal amount of DNA or siRNA with the empty plasmid pcDNA3 or negative control siRNA. The detection of both firefly and renilla luciferases activity were performed according to the Dual Luciferase Reporter Gene Assay Kit (Beyotime Biotechnology) 36 h post-transfection, relative luciferase activity (RLA) was calculated by normalizing to the renilla luciferase activity. Each experiment was repeated three times and the mean RLA was calculated for a statistical analysis.

### Immunoprecipitation

CHO-K1 or Hep-2 cells were cultured in 10-cm cell culture plates, next day CHO-K1 cells were co-transfected with the indicated plasmids, Hep-2 cells were mock infected or infected with HSV-1 (MOI = 1). 40 h post-transfection or post-infection, cells were washed with PBS and lysed for 30 min on ice in cold RIPA lysis buffer (NaCl, 150 mmol/L; NP-40, 1%; sodium deoxycholate, 0.5%; SDS, 0.1%; Tris-HCl, 50 mM pH 7.5). The cell lysate was centrifuged at 14,000 g for 10 min, and the supernatant was precleared by incubation with protein A+G agarose beads (Beyotime Biotechnology) for 1 h at 4°C. An equal aliquot of the cell lysates was reserved as an input sample, whereas the remaining lysates were incubated with anti-Cdk9, anti-CyclinT1, anti-ICP22 or anti-VP16 antibodies under standard immunoprecipitation conditions and incubated at 4°C for 3 h with gentle agitation. Normal rabbit or mouse IgGs were used as controls. Protein A-agarose beads were added to the protein-antibody mixture and incubated overnight at 4°C with rotation. The beads were collected by centrifugation and washed three times with RIPA buffer and then subjected to sodium dodecyl sulfate polyacrylamide gel electrophoresis (SDS-PAGE).

### Immunodepletion

CHO-K1 cell lysates were prepared as previously described. The cell lysates in RIPA buffer were incubated with 8 µg of Cdk9 and CyclinT1 antibodies or rabbit IgG (control) for 4 h at 4°C and then incubated with 100 µl of protein A+G sepharose beads overnight at 4°C. After centrifugation, the supernatants were collected and quantitated, and the protein supernatants of immunodepletion were subsequently subjected to immunoprecipitation or western blot analysis with the indicated antibodies.

### Western blot

The precipitated proteins were quantitated, and equal amounts of the protein supernatants were subjected to 10% SDS-PAGE electrophoresis and transferred to a PVDF membrane, followed by a 5% BSA-TTBS (BSA, 5%; Tris-HCl, 100 mM, pH 7.5; NaCl, 0.9%; Tween-20, 0.2%) block. The transferred membranes were subsequently treated with a specific primary antibody and a goat anti-mouse or anti-rabbit IgG-HRP secondary antibody. Chemiluminescene (ECL) detection of antigen-antibody complexes using Immobilon Western HRP Substrates (Millipore) was performed according to the standard protocol.

### Viral titration

Hep-2 cells were co-transfected with the indicated plasmids (pcDNA3 as control) for 30 h, then infected with HSV-1 at MOI of 1.5. 20 h post-infection, the medium from Hep-2 cells were collected to measure the virus titer by microtitration assay. Briefly, viral stocks were serially diluted 10 times, and 100 µL of the diluted stocks were added to Vero cells with 80%-confluent in each of the 96-well plates. Overall, 8 parallel wells for each dilution stock were used. The 96-well plates were incubated at 37°C in a 5% CO_2_ incubator for 5–7 d to observe the virus's cytopathicity. The 50% cell culture infective dose (CCID_50_) was determined by Reed-Muench assay.

### Quantitative RT-PCR

Total RNA was extracted from the infected Hep-2 cells using TRIzol reagent (Invitrogen) and reverse-transcribed to cDNA using the supermoIII RT kit (Bioteke, Beijing). Real-time PCR of ICP4, ICP0 and control β-actin mRNA was performed using the SYBR Premix Ex Tag GC (Takara) (Table S3). Relative ICP4 and ICP0 RNA levels were normalized to β-actin RNA levels, data were calculated as 2^−ΔΔCt^. All experiments were repeated three times and the mean value was calculated for the statistical analysis.

### Chromatin Immunoprecipitation (ChIP)

The chromatin immunoprecipitation procedure and quantification were performed as previously described with minor modifications [59]. Briefly, 1×10^8^ confluent CHO-k1 cells were co-transfected with different promoter reporter plasmids (pGL-α4, pGL-α4-Δ16, pGL-TK or pGL-VHS) in combination with the expression plasmids of ICP22 and/or VP16. For HSV-1 infected Hep-2 cells, 5×10^7^ Hep-2 cells were transfected with ICP22, VP16 or control pcDNA3 expression plasmids for 36 h, and then infected with HSV-1 at an MOI of 10 or 2 as indicated. 40 h post-transfection or 3–13 h post-infection, cells were treated with 2 mM EGS [Ethylene glycol-bis(succinic acid N-hydroxysuccinimide ester)] (Sigma-Aldrich) for 40 min to crosslink the proteins and then washed three times with PBS, followed by treatment with formaldehyde (FA) (1% final concentration) for 10 min at room temperature to crosslink proteins to DNA. Cells were then washed with cold PBS and lysed in SDS lysis buffer (SDS, 1%; EDTA, 10 mM; Tris–HCl, 50 mM pH 8.1). The lysate was sonicated to shear DNA to between 200 and 1000 bp. The sonicated supernatant was diluted 10-fold with the ChIP dilution buffer (SDS, 0.01%; Triton X-100, 1%; EDTA, 1.2 mM; Tris–HCl, 16.7 mM pH 8.1, NaCl 167 mM), precleared with salmon sperm DNA/protein A-agarose (Upstate) for 1 h, and then incubated with a rabbit polyclonal antibody against CyclinT1 (Santa Cruz) or the negative control IgG overnight at 4°C with rotation. Immune complexes were collected using salmon sperm DNA/protein A-agarose and washed sequentially for 5 min with low-salt buffer, high-salt buffer, LiCl buffer and TE buffer. The pellet was dissolved in elution buffer (1% SDS and 0.1 M NaHCO_3_) and centrifuged to remove the agarose. The supernatant was treated with 5 M NaCl and heated to 65°C for 8 h to reverse crosslinking. The immunoprecipitated DNA was purified by proteinase K treatment, phenol–chloroform extraction, and ethanol precipitation. Immunoprecipitated promoter DNA and input (non-immunoprecipitated) DNA were analyzed quantitatively by real-time PCR using the MightyAmp SYBR Plus reagent (Takara, Dalian, China) (Table S3). Relative immunoprecipitated DNA values were calculated as 2^−ΔΔCt^, where ΔΔCt = ΔCt (ICP22 and/or VP16)−ΔCt (pcDNA3), ΔCt (ICP22 and/or VP16) = Ct (IP)−Ct (input), and ΔCt (pcDNA3) = Ct (IP)−Ct (input). Each experiment was repeated three times and the mean value was calculated for the statistical analysis.

### Statistical Analysis

Data obtained from all experiments were described as the mean ± standard deviation (SD), and P<0.05 was considered to be statistically significant using a student's t-test.

## Supporting Information

Figure S1 Yeast trap and in vitro binding assay analysis of interaction between ICP22 and VP16. Yeast trap (upper panel): yeast strain AH109 was transformed with the indicated plasmids: pGAD-ICP22 and pGBK-VP16, negative control (pGADT7-T and pGBKT7-Lam), and positive control (pGADT7-T and pGBKT7-p53). Activity of β-galactosidase was measured respectively. The data represent relative β-galactosidase activity values were relative to the negative control from three independent experiments. In vitro binding assay (lower panel): 600 ng GST-VP16 fusion protein was incubated with 400 ng purified ICP22 protein at 4°C for 4 h (GST as control). 100 µl Glutathione-Sepharose precoated with BSA was then added and incubated overnight at 4°C. The protein-sepharose complexes were then boiled and subjected to 10% SDS-PAGE and analyzed by Western blotting using anti-ICP22 antibody.
(TIF)

Figure S2 The enhancement of P-TEFb binding to the transcription complex of viral α4 gene promoter in the presence of crosslinking agents in ChIP assays. CHO-K1 cells were transfected with pGL-α4 plasmid and subjected to ChIP assays. FA or FA plus EGS were used as the crosslinking agents. Antibodies specific for CyclinT1 or control rabbit IgG were used for immunoprecipitation. Real-time PCRs were performed to analyze the precipitated α4 promoter DNA. Values are expressed as percentages of input DNA immunoprecipitated. Error bars represented the standard deviation from triplicate samples.
(TIF)

Table S1 Primers for gene amplification. Oigonucleotide sequences for amplification of Cdk9 and CyclinT1 genes.
(DOC)

Table S2 Oigonucleotide sequences for RNA interference (RNAi). siRNA duplexes against Cdk9 and CyclinT1 genes.
(DOC)

Table S3 Primers for RT-PCR. Oligonucleotide sequences for quantitative real time PCR analysis.
(DOC)
