# Supplementary figures and images for: Herpes Simplex Virus 1 ICP22 Inhibits the Transcription of Viral Gene Promoters by Binding to and Blocking the Recruitment of P-TEFb
Source: PLoS One. 2012 Sep 24;7(9):e45749. doi: 10.1371/journal.pone.0045749 (PMC3454370; doi:10.1371/journal.pone.0045749)

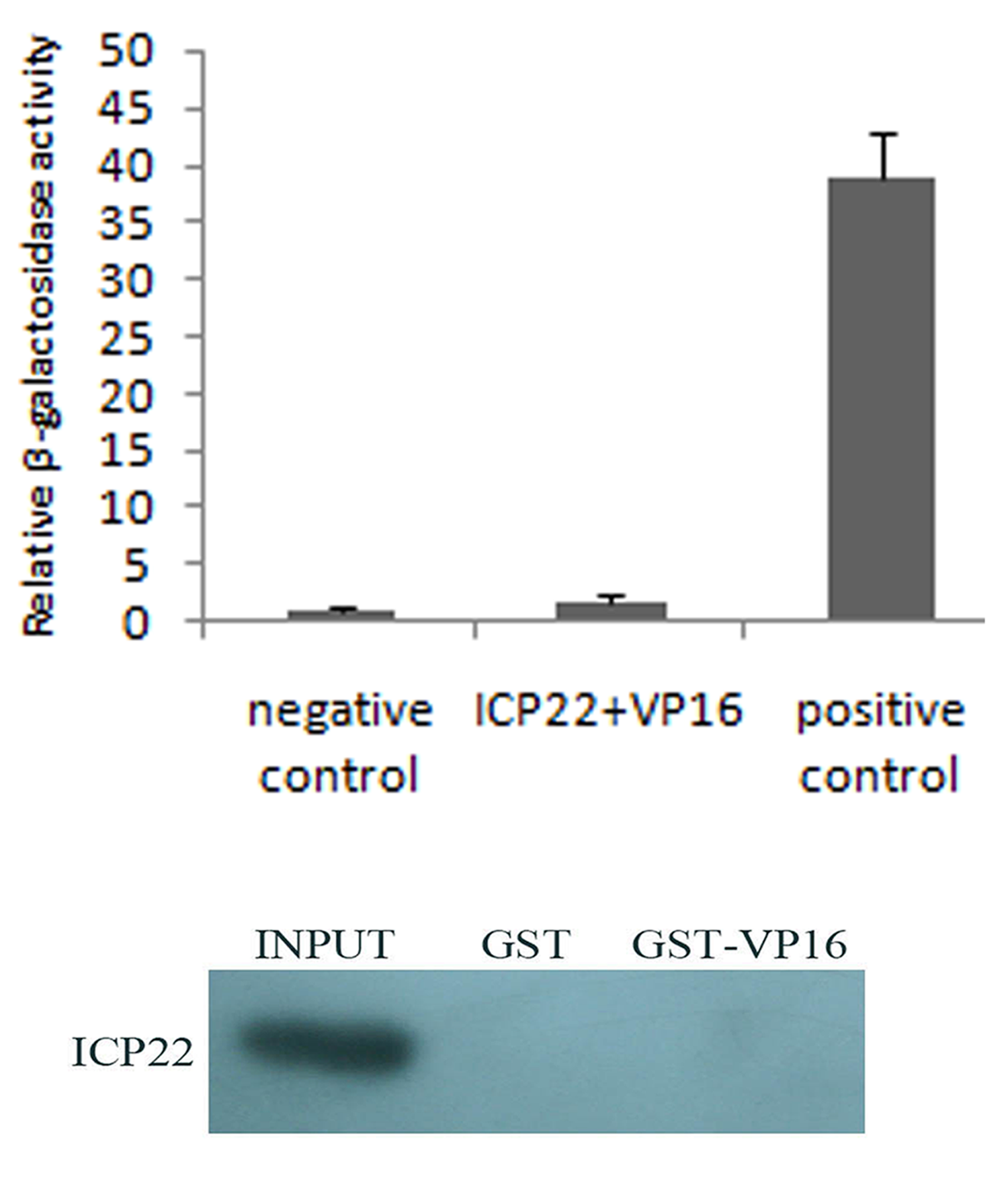

Supplement: Figure S1 — Yeast trap and in vitro binding assay analysis of interaction between ICP22 and VP16. Yeast trap (upper panel): yeast strain AH109 was transformed with the indicated plasmids: pGAD-ICP22 and pGBK-VP16, negative control (pGADT7-T and pGBKT7-Lam), and positive control (pGADT7-T and pGBKT7-p53). Activity of β-galactosidase was measured respectively. The data represent relative β-galactosidase activity values were relative to the negative control from three independent experiments. In vitro binding assay (lower panel): 600 ng GST-VP16 fusion protein was incubated with 400 ng purified ICP22 protein at 4°C for 4 h (GST as control). 100 µl Glutathione-Sepharose precoated with BSA was then added and incubated overnight at 4°C. The protein-sepharose complexes were then boiled and subjected to 10% SDS-PAGE and analyzed by Western blotting using anti-ICP22 antibody. (TIF) [file pone.0045749.s001.tif]

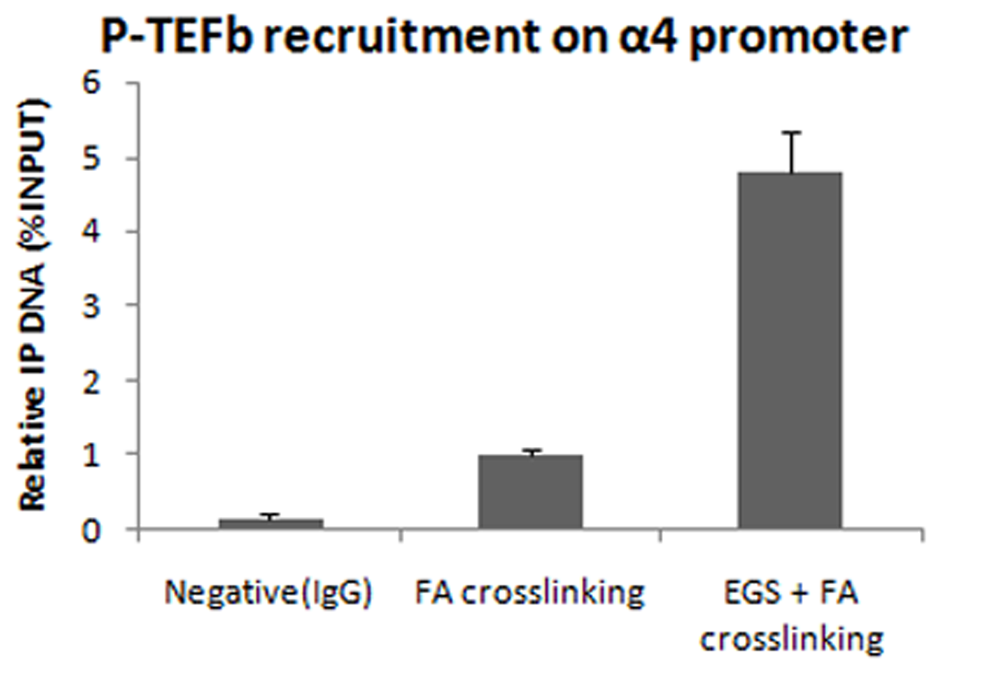

Supplement: Figure S2 — The enhancement of P-TEFb binding to the transcription complex of viral α4 gene promoter in the presence of crosslinking agents in ChIP assays. CHO-K1 cells were transfected with pGL-α4 plasmid and subjected to ChIP assays. FA or FA plus EGS were used as the crosslinking agents. Antibodies specific for CyclinT1 or control rabbit IgG were used for immunoprecipitation. Real-time PCRs were performed to analyze the precipitated α4 promoter DNA. Values are expressed as percentages of input DNA immunoprecipitated. Error bars represented the standard deviation from triplicate samples. (TIF) [file pone.0045749.s002.tif]
