# Supplementary material for: Herpes Simplex Virus 1 ICP22 Inhibits the Transcription of Viral Gene Promoters by Binding to and Blocking the Recruitment of P-TEFb
Source: PLoS One. 2012 Sep 24;7(9):e45749. doi: 10.1371/journal.pone.0045749 (PMC3454370; doi:10.1371/journal.pone.0045749)
Supplement: Table S1 — Primers for gene amplification. Oigonucleotide sequences for amplification of Cdk9 and CyclinT1 genes. (DOC) [file pone.0045749.s003.doc]

**Table S1.** Oigonucleotide sequences for amplification of Cdk9 and CyclinT1 gene

| **Amplicon**  **Name** | **Forward Primer** | **Reverse Primer** |
| --- | --- | --- |
| Cdk9 | ATGGCAAAGCAGTACGACTC | TCAGAAGACGCGCTCAAACTC |
| CyclinT1 | ATGGAGGGAGAGAGGAAGAAC | CTTAGGAAGGGGTGGAAG |
