# Supplementary material for: Herpes Simplex Virus 1 ICP22 Inhibits the Transcription of Viral Gene Promoters by Binding to and Blocking the Recruitment of P-TEFb
Source: PLoS One. 2012 Sep 24;7(9):e45749. doi: 10.1371/journal.pone.0045749 (PMC3454370; doi:10.1371/journal.pone.0045749)
Supplement: Table S2 — Oigonucleotide sequences for RNA interference (RNAi). siRNA duplexes against Cdk9 and CyclinT1 genes. (DOC) [file pone.0045749.s004.doc]

**Table S2.** Oigonucleotide sequences for RNA interference (RNAi)

| **siRNA** | **Oligo** |
| --- | --- |
| Si-Cdk9 | CCAAAGCUUCCCCCUAUAAdTdT |
| Si-CyclinT1 | UCCCUUCCUGAUACUAGAAdTdT |
