# Supplementary material for: Herpes Simplex Virus 1 ICP22 Inhibits the Transcription of Viral Gene Promoters by Binding to and Blocking the Recruitment of P-TEFb
Source: PLoS One. 2012 Sep 24;7(9):e45749. doi: 10.1371/journal.pone.0045749 (PMC3454370; doi:10.1371/journal.pone.0045749)
Supplement: Table S3 — Primers for RT-PCR. Oligonucleotide sequences for quantitative real time PCR analysis. (DOC) [file pone.0045749.s005.doc]

**Table S3.** Oligonucleotide sequences for quantitative Real Time PCR analysis

| **Amplicon**  **Name** | **Forward Primer** | **Reverse Primer** |
| --- | --- | --- |
| α4 promoter | GGTAATGAGATGCCATGCGG | TCGGGCTCATATAGTCCCAG |
| TAATGARAT-deleted α4 promoter | AGCAGGCTGTGGCTCTGATT | CAAAATAGCCACCAGCCTCTTCT |
| TK promoter | TATCTTGTCACCCGGAGGCG | CCGACTGCATCTGCGTGTTC |
| VHS promoter | TCGCTCACCGAGCCAGAAAC | TTCGATGGCCCAACTCCACG |
| α4 gene | GCAGCAGTACGCCCTGA | TTCTGGAGCCACCCCATG-3 |
| ICP0 gene | GTGCATGAAAACCTGGATGC | TTGCCCGTCCAGATAAAGTC |
| β-actin gene | GGCATCCTCACCCTGAAGTA | GGGGTGTTGAAGGTCTCAAA |
